# Human Epidermal Growth Factor Receptor-2 gene polymorphism and breast cancer risk in women from the Northeastern region of Brazil

**DOI:** 10.6061/clinics/2020/e2360

**Published:** 2020-11-26

**Authors:** Carla Solange Escórcio-Dourado, Francisco Adelton Alves-Ribeiro, Jose Charles Lima-Dourado, Alesse Ribeiro dos Santos, Renato de Oliveira Pereira, Cleciton Braga Tavares, Vladimir Costa Silva, Pedro Vitor Lopes Costa, José Maria Soares-Júnior, Benedito Borges da Silva

**Affiliations:** I Programa de Pos-Graduacao da Rede Nordeste de Biotecnologia (RENORBIO), Nordeste, Universidade Federal do Piaui, Teresina, PI, BR.; II Programa de Pos-Graduacao em Ciencias Medicas, Universidade Federal do Piaui, Teresina, PI, BR.; III Departamento de Obstetricia e Ginecologia, Hospital das Clinicas (HCFMUSP), Faculdade de Medicina, Universidade de Sao Paulo, SP, BR.

**Keywords:** Breast Cancer Risk, Genetic Polymorphism, HER2 rs1136201 Variant, Real-Time PCR

## Abstract

**OBJECTIVES:**

In the Human Epidermal Growth Factor Receptor-2 (HER2) rs1136201 variant, the presence of the G allele may promote cellular alterations and increase breast cancer risk, in addition to enhanced cellular proliferation, tumor aggressiveness, and metastases. The aim of this study was to investigate the presence of the single-nucleotide polymorphism (SNP) variant, rs1136201, within the HER2 gene in women from the Northeastern region of Brazil and breast cancer risk.

**METHODS:**

The study included 140 women who were divided into two groups, case (breast cancer) and control (without breast cancer), with 70 women in each group. Peripheral blood of each woman was drawn for the study of genomic Deoxyribonucleic acid (DNA) extracted from leukocytes using the genotyping technique by real-time polymerase chain reaction.

**RESULTS:**

The GG genotype occurred in 1 woman in both groups (1.4%) (*p*=0.32), while the AG genotype occurred in 19 (27.2%) and 13 (18.6%) women in the case and control (*p*=1.00) groups, respectively. No statistically significant difference in GG and AG genotypes was observed between the case and control groups in premenopausal women (*p*=1.00). Furthermore, no significant difference in genotypes was observed between the groups, among postmenopausal women (*p*=0.14).

**CONCLUSION:**

In this study, the HER2 rs1136201 polymorphism did not show any statistically significant association with breast cancer, both in premenopausal and postmenopausal women. Nevertheless, further studies with a larger sample size should be performed to assess the association of HER2 polymorphism with breast cancer risk in women from the Northeastern region of Brazil.

## INTRODUCTION

Breast cancer is a neoplasm that most commonly affects women worldwide, with an estimate of 2,088,849 new cases and 626,679 deaths in 2018 based on GLOBOCAN 2018 (1). Breast cancer incidence is higher in the most developed country of the world compared to underdeveloped countries (2,3). In Brazil, following non-melanoma skin cancer, breast cancer is the most common malignancy in women, with an estimate of 59,700 new cases and 16,927 deaths in 2019 (4). Further, the Brazilian National Cancer Institute estimates approximately 66,280 new cases of breast cancer in Brazilian women for 2020 (5). Although physical examination and mammography are important for early diagnosis and to reduce mortality, in underdeveloped and developing countries such as Brazil, the disease is commonly diagnosed in advanced stages, resulting in high mortality rates (6-8). Despite early diagnosis and current therapeutic strategies that are important to reduce mortality, the estimate of breast cancer deaths in Brazil for 2020 is 17,572 cases (5).

Breast cancer is a disease of unknown etiology that involves multiple risk factors of which genetic alterations are the major ones (9). Alterations of human epidermal growth factor receptor 2 (HER2) proto-oncogene (also known as erbB-2 or HER2/neu) have been implicated in the carcinogenesis and prognosis of breast cancer; they have also been thought to play a critical role in both breast cancer development and progression (10,11). Siddig et al. (12) have shown the association of breast cancer risk with single nucleotide polymorphism (SNP) in HER-2/neu, Ile655Val [db SNP rs1136200]. ERBB2 is a proto-oncogene, that is present at higher levels in premenopausal women and in estrogen receptor (ER) negative breast cancers than in ER positive breast cancers (13). The HER2 gene, located on chromosome 17q21, is a member of the human epidermal growth factor receptor family. Breast cancers that overexpress HER2 are an aggressive molecular subtype, exhibiting rapid tumor growth, increased risk of postoperative recurrence, resistance to hormone therapy, and poor response to conventional breast carcinoma chemotherapy (13).

SNP in the HER2 gene is one of the most important genetic alterations in breast cancer (14). Various studies on genotyping have correlated HER2 Ile655Val (rs1136201 variant) polymorphism and breast cancer risk (10-12), while other authors have not shown an increased risk for breast cancer in women with HER2 Ile655Val (rs1136201) polymorphism (15). Thus, the results have been inconsistent and conflicting, despite the fact that the majority of authors have suggested a positive association between the presence of HER2 rs1136201 polymorphism and risk of breast cancer development (10-12,14). These controversies over the HER2 rs1136201 polymorphism and breast cancer risk motivated us to design the current study.

## MATERIAL AND METHODS

### Patients and blood samples

The Internal Review Board of the Federal University of Piaui approved the study under number 43447015.8.0000.5214 and all patients signed an informed consent form prior to admission. This is a controlled cross-sectional study, involving patients managed in the Breast Disorders Outpatient Clinic of the Getulio Vargas (HGV) Hospital and Laboratory of Molecular Biology of the Natan Portella Hospital, Federal University of Piaui, Brazil, from March 2016 to December 2018. The study included 140 women, divided into two groups of 70 women each: case group (with breast cancer) and control group (without breast cancer). In fact, considering the asymptotic test to compare two sample proportions if the true proportions were *p*1=0.32 and *p*2=0.2, the sample sizes would be n1=n2=70, with a 5% level of significance. The power of the two-sided test would be only 37% and a on-sided test would have a statistical power of 49%. The low power is the main caveat of this study. Patients with histologically confirmed breast cancer and healthy patients (controls) that were negative for malignancy were included in the sutdy, as confirmed by physical examination and imaging tests. Women older than 80 years and patients suffering from liver, metabolic, cardiovascular, kidney disease or those reporting other types of malignancies were excluded from the study. A volume of 3 mL of blood was drawn using disposable syringes and needles by a specialized technician, after the patient received medical consultation. Whole blood was stored in proper vials containing anticoagulant (EDTA) and stored in a freezer at −20 °C. All women in the study were instructed not to use anti-inflammatory drugs during the 72 h before blood sample collection.

### DNA extraction

For DNA extraction of sample leukocytes, the PureLink Genomic^®^ DNA Mini Kit (Life Technologies) was used, following manufacturer’s instructions.

### Genotyping

After isolation, DNA concentration was determined by Nanodrop2000 spectrophotometer (Thermo Fisher Scientific, Waltham, MA, USA). Genotyping was performed by real-time polymerase chain reaction (qPCR). Absolute DNA quantification of a sample is performed with the use of a standard curve, obtained by amplification of known quantities of the same DNA. SNP genotyping assays contain a probe labeled with VIC^®^ dye and a probe labeled with FAM^TM^ dye. TaqMan^®^ probes incorporate minor groove (MGB) binder technology, where the VIC^®^ probe detects allele 1 and the FAM^TM^ probe detects allele 2. The reactions were conducted in final volumes of 20 μL per patient, containing: 10 μL TaqMan^®^ Genotyping Master Mix; 0.5 μL of TaqMan^®^ probe customized for SNP genotyping of HER2 human gene (SNP ID rs1136201.Cod.C___7452451_1_Context sequence VIC/FAM (5' fluorescent reporter dye):CGCCCCCAGCCCTCTGACGTCCATC[A/G]TCTCTGCGGTGGTTGGCATTCTGCT) (Table 1); 5.5 µL deionized DNA/RNA-free water and 4 µL DNA sample per patient; these volumes were distributed in 96-well reaction plates (MicroAmp_ Fast Optical 96-Well Reaction Plate) with 0.1 mL per plate (Applied Biosystems, EUA) and in duplicates. Amplification was performed using the Fast Real-Time PCR System 7500 with the SDS 2.2 software incorporated for SNP genotyping (Applied Biosystems, EUA) in the following steps: (1) pre-PCR step for 1 min at 60 °C; (2) pre-incubation of the reaction mixture at 95 °C for 10 min; (3) thermocycling at 95 °C for 15 s and 60 °C for 60 s for 40 cycles; and (4) post-PCR step for 1 min at 60 °C. Fluorescence data were captured during 40 reaction cycles. Quality control of qPCR was assessed by random selection of 20% of the total samples for re-genotyping by an independent technician.

### Statistical analysis

The Chi-square test was used to determine whether genotype distribution conformed to Hardy-Weinberg equilibrium. Genotype frequency was compared between women with breast cancer and women without the disease from a control group, using Fisher’s exact test. The odds ratio (OR) and 95% confidence interval (CI) were calculated using Fisher’s exact test due to low frequencies in lines. Statistical significance was set at *p*<0.05.

## RESULTS

The study included 140 women (70 cases and 70 controls). Mean patient age and standard deviation was 49.1 ± 11.1 years for cases and 45.4 ± 12.8 for controls. Genotype frequencies of the rs1136201 variant of the HER2 gene conformed to Hardy-Weinberg equilibrium. The GG genotype was present in one woman (1.4%) in the case and control groups (*p*=0.32), while the AG genotype occurred in 19 (27.2%) women from the case group and in 23 (32.9%) women from the control group (*p*=1.00) (Table 2). After stratification according to menopausal status, no statistically significant difference in GG and AG genotypes was observed between the case and control groups in premenopausal women (*p*=1.00). In addition, no significant difference was found between postmenopausal women from the case and control groups (*p*=0.14) (Table 3).

## DISCUSSION

The Brazilian population is constituted by a rich racial miscegenation, mainly composed of European descendants, African and indigenous populations. Therefore, as in other countries with a different racial pattern, the distributions of genetic variants in the Brazilian population commonly do not show a consistent genetic pattern (9). HER2 gene is found amplified in tumors and some authors showed that single nucleotide polymorphism at codon 655 (Ile655Val) is not associated with an increase in breast cancer risk (16), while other authors suggest that HER2 Ile 655Val polymorphism may contribute to breast cancer risk (17). In addition to these controversies, to the best of knowledge, there is a scarcity of studies on HER2 gene polymorphism, particularly its rs1136201 variant, associated with breast cancer risk in women from Northeastern Brazil. The current study did not show statistically significant differences between genotypes of case and control groups. It also did not show any differences in premenopausal and postmenopausal women for the rs1136201 variant of the HER2 gene between the above-mentioned groups.

In cancer, HER2 may be deregulated by a variety of mechanisms, including mutations, HER2 polymorphism, HER2 overexpression, and overproduction of ligands for HER2 activation (18). Analyses of human DNA clones identified polymorphism at codon 655 in the transmembrane coding region of the HER2 gene, which encodes isoleucine (Ile; ATC) or valine (Val; GTC) and has been reported in different types of cancer. The presence of Val (G) in the transmembrane position stabilizes the formation of an active dimer of the protein that predisposes HER2 to auto-activity (19). In addition, Takano et al. (20) suggested that the Ile (A) to Val (G) that change in the HER2 codon 655 may alter hydrophobicity of the HER2 protein, affecting the conformational stability of the hydrophobic domains within the transmembrane domain. Likewise, some authors have suggested that the presence of this polymorphism in cancer enhances dimerization, self-phosphorylation, and HER2 tyrosine kinase activity, which may cause cell transformation (21).

A case-control study of HER2 Ile655Val including Chinese women suggested that the GG homozygote was associated with an increased risk for breast cancer (10). Lee et al. (22) investigated the HER2 Ile655Val SNP in Taiwanese women with early breast cancer and suggested that the presence of GG genotype may be a risk factor for early-onset breast cancer. Likewise, other authors have suggested that the presence of GG homozygote may be a risk factor for breast cancer in Caucasian populations and in African women (23,24). Ma et al. (25) showed that the heterozygous AG genotype had a modest association with breast cancer risk. Nevertheless, some authors show that the SNP rs1136201 of the HER2 gene was not associated with an increased breast cancer risk (16); corroborating these findings, Breyer et al. (26) did not observe any association between this SNP and breast cancer risk.

Thus, some authors hypothesized that the HER2 Ile655Val polymorphism may be considered a genetic biomarker of susceptibility to breast cancer risk, although it is not reliable for the estimation of tumor aggressiveness. However, the association between the HER2 Ile655Val (rs1136201) SNP and breast cancer risk has still not been fully elucidated (27). Furrer et al. (28) studied the association between this polymorphism and non-metastatic HER2-positive breast cancer; they theorized that the HER2 Ile655Val SNP may play a significant role in breast carcinogenesis in Caucasian women. In contrast, Watrowski et al. (15) did not show any association between this SNP and susceptibility to breast cancer in Caucasian women.

Several authors have shown conflicting results regarding the association between the HER2 and breast cancer; HER2 has somatic variations in different ethnicities (6). Our results are in agreement with Ile655Val SNP and breast cancer risk (15,16,26). Nevertheless, it is important to bear in mind that these studies have been conducted in different ethnic groups with genetic polymorphisms composed of descendants of European, African and Asian with different genetic patterns (9). Likewise, in Brazil, it was not shown presence of significant difference of HER2 polymorphism between breast cancer women and controls, probably due to racial widespread miscegenation without a consistent genetic pattern and also sample size.

## CONCLUSIONS

Therefore, in this study, there was no significant association observed between the SNP rs1136201 variant of the HER2 gene and breast cancer risk in both premenopausal and postmenopausal women from the Northeastern region of Brazil. Nevertheless, owing to the high degree of racial miscegenation in Brazil, further studies with a higher statistical power and a larger sample size are warranted.

## AUTHOR CONTRIBUTIONS

Escórcio-Dourado CS, Alves-Ribeiro FA, Lima-Dourado JC, da Silva BB conceived the study idea, interpreted the data, wrote and revised the manuscript. dos Santos AR, de Oliveira Pereira R, Tavares CB participated in the design, coordination of the study, interpreted the data, wrote and revised the manuscript. Silva VC, Costa PV collected the materials and conducted data extraction. Soares-Júnior JM, da Silva BB analyzed, interpreted the data and revised the manuscript. All authors have read and approved the manuscript.

## Figures and Tables

**Table 1:** Identification of gene codes and SNP used in TaqMan® assay Source Life Technologies.

Gene variant HER2	Sequence context VIC/FAM
rs1136201 A>G	CGCCCCCAGCCCTCTGACGTCCATC[A/G]TCTCTGCGGTGGTTGGCATTCTGCT

**Table 2:** Patient characteristics and genotyping of HER2 gene SNP rs1136201 in case and control patients.

Characteristics	Case	Control		
N	70	70		
Mean age (SD)	49.1 (±11.1)	45.4 (±12.8)		
Premenopausal	39	33		
Postmenopausal	31	37		

	Case (%)	Control (%)	p	OR (95% CI)
rs1136201				
AA	50 (71.4)	46 (65.7)	-	1
AG	19 (27.2)	23 (32.9)	1	1.08 (0.34-3.4)
GG	1 (1.4)	1 (1.4)	0.32	1.59 (0.68-3.81)

There was no statistically significant difference of SNP genotype rs1136201 between case and control groups (*p*>0.05). The odds ratio (OR) was not significantly higher between groups.

**Table 3:** Genotyping of HER2 gene SNP rs1136201 in premenopausal and postmenopausal women.

	Premenopausal				Postmenopausal			
	Case	Control	*p*	OR (95% CI)	Case	Control	*p*	OR (95% CI)
AA	30	26	-	1	20	20	-	1
A/G	8	6	0.7	1.1 (0.34-3.4)	11	17	0.14	2.7 (0.6-13.6)
GG	1	1	1	1.4 (0.23-4.3)	0	0	-	-

There was no statistically significant difference between SNP genotypes rs1136201 in the case group and control group (*p*>0.05), as well as in the postmenopausal group (*p*>0.05). There was no significantly higher odds ratio (OR).
